# Verification of harmonized reference intervals in Croatia: is it time for a change?

**DOI:** 10.11613/BM.2026.020706

**Published:** 2026-06-15

**Authors:** Iva Friščić, Ida Burazer, Andrea Radeljak, Sonja Perkov, Mirjana Mariana Kardum Paro

**Affiliations:** 1 Department of Medical Biochemistry and Laboratory Medicine, Merkur University Hospital, Zagreb, Croatia; 2 Department for Clinical Laboratory Diagnostics, Children’s Hospital Srebrnjak, Zagreb, Croatia

**Keywords:** reference values, verification, harmonization, CLSI

## Abstract

**Introduction:**

The study of harmonized reference intervals (RIs) in Croatia was published in 2004. Since then, notable methodology and population characteristics changes have occurred in the Croatian population, highlighting the need for re-evaluation. This study aimed to verify harmonized RIs for hematology, coagulation, and clinical chemistry laboratory tests and assess their applicability in one clinical hospital center.

**Materials and methods:**

This verification study included 100 apparently healthy adults (40 males, 60 females; aged 22 to 75 years), selected using both *a priori* and *a posteriori* exclusion criteria following CLSI EP28-A3c guidelines. Blood samples were collected under standardized preanalytical conditions. Hematology analysis was performed on the Sysmex XN1000 analyzer (Sysmex, Kobe, Japan), coagulation testing on the Sysmex CS2500 analyzer (Siemens Healthcare Diagnostics Inc., Marburg, Germany), and clinical chemistry analysis on the Beckman Coulter DxC700AU (Beckman Coulter, Brea, USA) and Radiometer ABL90 FLEX (Radiometer, Brønshøj, Denmark) analyzers. Reference intervals were considered verified if at least 90% of results (18/20) fell within the predefined RIs.

**Results:**

All tested hematology and coagulation RIs were successfully verified. Among 27 tested biochemical analytes, 25 RIs were verified in the first sample set. Total calcium and alkaline phosphatase RIs required additional verification. International harmonized RIs were adopted and successfully verified for both tests using two independent sample sets.

**Conclusions:**

Most harmonized RIs in use in Croatia remain applicable to the adult population of one clinical hospital center. However, periodic re-evaluation is needed due to changes in analytical methods and population characteristics. Reference intervals from international harmonization projects can provide a valid alternative when local verification fails.

## Graphical abstract

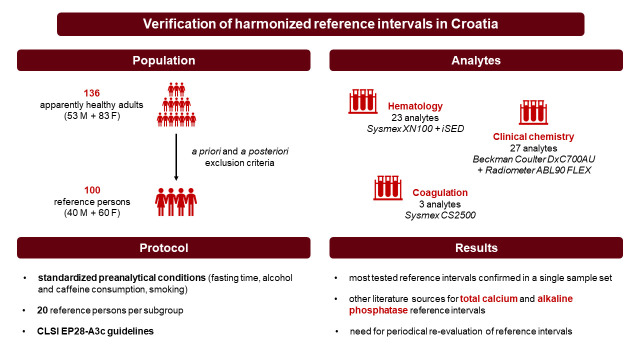

## Highlights

• Harmonized reference intervals were verified in healthy adults in Croatia

• Most tested reference intervals were confirmed in a single sample set

• International harmonized reference intervals were adopted for total calcium and alkaline phosphatase

• Periodic re-evaluation of harmonized reference intervals is needed

## Introduction

Reference intervals (RIs) are a fundamental tool for the assessment of laboratory test results. The use of appropriate RIs provides clinicians with the proper context for interpreting laboratory test results and has a significant impact on the clinical decision-making process (*1*, *2*). The establishment of direct RIs is a time-consuming and financially demanding process, and the derivation of indirect RIs requires advanced informatics infrastructure and expertise (*1*-*4*). Furthermore, the derivation of indirect RIs has additional limitations related to reference population selection and the exclusion of pathological results, as well as to the standardization of preanalytical conditions and analytical methods (*1*, *4*, *5*). Consequently, most laboratories routinely transfer and verify RIs from literature sources, which mostly include manufacturers’ test instructions, national or international expert group recommendations, and harmonized RIs derived using direct single- or multicenter studies or indirect approaches (*1*, *2*, *4*-*7*). In the context of harmonization, international initiatives coordinated by the International Federation of Clinical Chemistry and Laboratory Medicine (IFCC) have played a key role. The IFCC Committee on Reference Intervals and Decision Limits (C-RIDL) and the IFCC Task Force on Global Reference Interval Database (TF-GRID) have emphasized the use of standardized protocols, multicenter data, and large-scale datasets to improve the comparability and transferability of RIs across populations and analytical platforms. The primary objective of the TF-GRID is the development of an IFCC-hosted platform serving as a centralized global RI database for clinical chemistry and hematology (*1*, *2*, *4*, *5*). Despite these efforts, local verification remains essential before routine implementation due to population- and laboratory-specific factors (*1*). In Croatia, the national RI harmonization project was initiated in 2003 by the Croatian Chamber of Medical Biochemists (CCMB). It resulted in publications released in 2004 and 2007, which include RIs in general, specialized and highly specialized medical biochemistry (*8*, *9*). These RIs were based on data obtained from the population of Zagreb and its surrounding area, whose demographic characteristics most closely align with those of the Croatian population (*10*). Samples used for the establishment of RIs were collected across multiple centers, while laboratory analyses and statistical data processing were performed in a single center, which also serves as the national Reference Centre for the development and implementation of RIs. Current national recommendations are primarily based on previously published data (*8*-*10*). For the pediatric population, the use of RIs from international literature sources is recommended, primarily those from the Canadian Laboratory Initiative on Pediatric Reference Intervals database (CALIPER database) (*11*). Following the initial publications, verification of RIs was performed for serum creatinine (CREA) measured using the enzymatic method, as well as for aspartate aminotransferase (AST) and alanine aminotransferase (ALT), whose catalytic activities are determined using the IFCC reference methods with pyridoxal-5-phosphate (PLP) activation. Given the high degree of standardization of these methods, the internationally harmonized RIs are considered suitable for the studied population (*12*, *13*). The study by Flegar-Meštrić *et al*., published in 2016, represents the most recent publication in Croatia to provide a critical review of harmonized RIs for the adult population (*13*). Verification of RIs for the neonatal population in Croatia was conducted in 2024, demonstrating minimal differences compared to the evaluated RIs (*14*). Since the last verification of RIs for the adult population (*12*, *13*), significant methodological, dietary, and demographic changes have occurred within the Croatian population. On this basis, we aimed to verify the harmonized RIs for routine hematology, coagulation, and clinical chemistry laboratory tests in adults aged 20 years and older currently in use in clinical laboratories in Croatia (*8*, *9*, *13*). To this end, a single-center university hospital with a large urban catchment area, serving approximately one fifth of the population of the city of Zagreb, Croatia, provided the case study for this investigation.

## Materials and methods

### Subjects

This study was approved by the Ethical Committee of Mekur University Hospital, Zagreb, Croatia (approval number: 03/1-4884/4). All subjects signed an informed consent form. The study was conducted between July 2024 and June 2025. The study population consisted of adults aged 20 years and older. Reference individuals were selected from a larger group of apparently healthy volunteers recruited from hospital staff and the general population, using both *a priori* and *a posteriori* sampling methods. The *a priori* selection included the use of a standardized questionnaire developed following the international CLSI EP28-A3c guidelines, designed to assess medical history, lifestyle factors, and medication use (*15*). Exclusion criteria included self-reported disease, regular alcohol consumption, the use of addictive substances, and blood sampling under non-fasting conditions. Self-reported disease was defined as a positive response to questionnaire items 6-9. In addition, hypertension was considered an exclusion criterion when it required regular medication, as indicated by a positive response to questionnaire items 11 and 12. Alcohol consumption was assessed by self-report, where “occasional” referred to infrequent, social alcohol intake, while “regular” consumption indicated habitual alcohol use; individuals reporting regular consumption were excluded. The questionnaire is shown in Table 1.

**Table 1:** Standardized questionnaire used for selection of reference individuals

**Number**	**Question**	**Answer format**
1	Full name	Free text
2	Age	Numeric
3	Sex	Male / Female
4	Height (cm)	Numeric
5	Weight (kg)	Numeric
6	Do you consider yourself a healthy person?	Yes / No
7	Do you suffer from any chronic diseases?	Free text
8	Have you been hospitalized in the past 3 months?	Yes (specify) / No
9	Have you had any serious illnesses in the last 3 years?	Yes (specify) / No
10	Hereditary or chronic diseases in the family	Yes (specify) / No
11	Hypertension	Yes / No
12	Regular medication, including dietary supplements	Free text
13	Physical activity	Regular (≥ 3x/week) / Occasional / Never
14	When did you last exercise?	Free text
15	Smoking	Yes / No
16	Alcohol consumption	Never / Occasionally / Regularly
17	Use of addictive substances	Yes / No
18	Special diet	Free text
19	Other	Free text
20	Blood sampling in accordance with professional guidelines (filled by lab staff)	Yes / No

*A posteriori* exclusion criteria included the presence of an acute inflammatory process, indicated by elevated C-reactive protein (CRP) concentration (> 5 mg/L) and total leukocyte count (> 9.4×10^9^/L), and specific reasons stated under the questionnaire item “Other”. For certain laboratory tests, specific exclusion criteria were applied, including the use of medication and dietary supplements for liver enzymes and total and direct bilirubin concentrations, and physical exercise within three days before blood sampling for creatine kinase (CK). Serum and plasma samples with hemolysis, icterus, or lipemia index (HIL) ≥ 1 were also excluded.

### Methods

The verification was conducted at the Department of Medical Biochemistry and Laboratory Medicine, Merkur University Hospital, Zagreb, Croatia, designated as the national Reference Centre for the development and implementation of RIs. All analytical methods used in this study are accredited according to the International Organization for Standardization (ISO) standard 15189:2022, which requires continuous internal quality control and participation in external quality assessment schemes (*16*).

Blood sampling was performed following the Joint EFLM-COLABIOCLI Recommendation for venous blood sampling (*17*). All samples were collected between 7:00 and 9:00 a.m. After completing the questionnaire, each subject was identified by stating their full name and date of birth. The phlebotomist verified and recorded the fasting status, which required at least 12 hours without food intake, no alcohol consumption within the previous 24 hours, and refraining from caffeine-containing beverages and smoking on the morning of blood collection. For each subject, three blood tubes were collected. Blood for hematology analysis was drawn into K_3_EDTA vacuum tubes (BD Vacutainer K_3_EDTA, K3E, Franklin Lakes, USA), blood for coagulation analysis into sodium citrate vacuum tubes (3.2% [0.109 M], BD Vacutainer 9NC, Franklin Lakes, USA), and blood for clinical chemistry analysis into red-cap vacuum tubes containing micronized silica particles as a clot activator, without gel (BD Vacutainer Serum, clot activator, no gel, Franklin Lakes, USA). Blood collection followed the recommended order of draw: citrate tube-clot activator tube-EDTA tube. Hematology analysis was performed on whole blood within 30 minutes of blood collection. Vacuum tubes intended for coagulation and clinical chemistry analyses were centrifuged before analysis at 2100×g for 15 and 10 minutes, respectively. All analyses were completed within 90 minutes of blood collection. The serum sample for clinical chemistry analysis was divided into two aliquots: one was immediately analyzed on the blood gas analyzer Radiometer ABL90 FLEX (Radiometer, Brønshøj, Denmark) for ionized calcium, while the other was analyzed for all remaining analytes on the clinical chemistry analyzer Beckman Coulter DxC700AU (Beckman Coulter, Brea, USA). Hematology analyses were performed on the Sysmex XN1000 analyzer (Sysmex, Kobe, Japan), erythrocyte sedimentation rate (ESR) was measured on the iSED analyzer (Alcor Scientific, Smithfield, USA), and coagulation testing was performed on the Sysmex CS2500 analyzer (Siemens Healthcare Diagnostics Inc., Marburg, Germany). For each sample, HIL indices were determined using a Beckman Coulter photometric test and assessed semi-quantitatively based on the approximate concentration of the interferent, with HIL ≥ 1 corresponding to ≥ 0.5 g/L hemoglobin, ≥ 0.025 g/L (42,8 µmol/L) bilirubin, or ≥ 0.4 g/L Intralipid, according to the Beckman Coulter manufacturer’s instructions for use. Detailed information on analytical methods, reagents, instruments, and traceability is provided in Supplementary Table 1.

### Statistical analysis

Reference interval partitioning by age and/or sex for individuals aged 20 years and older was performed according to the harmonized RIs currently in use in Croatia (*8*-*10*, *12*, *13*). According to international CLSI EP28-A3c guidelines, RI verification was performed using 20 reference individuals *per* subgroup (*15*). For age- and sex-independent RIs, the sample set included 10 males and 10 females. A RI was considered verified if at least 18 out of 20 results (90%) fell within the predefined RI. If this criterion was not met in the first set, a second set of 20 samples was analyzed under the same conditions. If the criterion was still not met, RIs from alternative literature sources were considered for further verification. All data were tabulated and analyzed using Microsoft Excel (Microsoft Corporation, Redmond, USA) and MedCalc for Windows, version 17.9.2 (MedCalc Software, Ostend, Belgium).

## Results

Baseline characteristics of the study population are summarized in Table 2. A total of 136 individuals presented for blood sampling. Out of these, 53 (39%) were male, and 83 (61%) were female. The age range was 26 to 75 years for men and 22 to 68 years for women. Applying the *a priori* exclusion criteria, 2 male and 4 female individuals were excluded due to self-reported chronic diseases and hospitalization within the previous 3 months. An additional 11 male and 19 female individuals were excluded based on *a posteriori* exclusion criteria. One male participant was excluded for a reason stated under the questionnaire item “Other” (voluntary blood donation within 30 days before blood sampling), while all others were excluded due to parameters indicating an acute inflammatory process. Due to specific exclusion criteria for liver enzymes and bilirubin, which included the use of medication and dietary supplements, 1 male and 6 female individuals were excluded. Based on the CK-specific exclusion criterion involving physical exercise within 3 days before blood sampling, 13 male and 18 female individuals were excluded. Analyte-specific exclusion criteria resulted in minor differences in age distribution. No samples had hemolysis, lipemia, or icterus index ≥ 1.

**Table 2:** Baseline characteristics of the study population used for reference interval verification

	**Initial population**	**Final RI population**	Bilirubin/liver enzymes population*	**CK population^†^**
Total	136	100	93	69
M, N (%)	53 (39)	40 (40)	39 (42)	27 (39)
F, N (%)	83 (61)	60 (60)	54 (58)	42 (61)
Age range, years (M)	26-75	26-75	26-75	29-75
Age range, years (F)	22-68	22-68	22-68	25-68
*Exclusion based on medication and dietary supplement use. ^†^Exclusion based on physical exercise within 3 days before blood sampling. M - male. F - female. RI - reference interval. CK - creatine kinase.				

### Hematology reference intervals

Reference intervals for red blood cells (RBC), hemoglobin (Hb), and hematocrit (Hct) are sex-dependent, while RIs for erythrocyte sedimentation rate (ESR) are both age- and sex-dependent (with separate intervals for males and females under and over 50 years of age). All other hematology RIs are age- and sex-independent. The sample set for age- and sex-independent RIs included 10 male and 10 female individuals, aged 22 to 75 years. Male-specific RIs included individuals aged 26 to 75 years (age-independent), 26 to 48 years (under 50), and 50 to 75 years (over 50), while female-specific RIs included individuals aged 22 to 68 years (age-independent), 22 to 46 years (under 50), and 51 to 68 years (over 50). All tested RIs were confirmed in the first set of samples. Verification results for all analytes, including RI partitioning, current RIs, sample size, median, interquartile range (IQR), and the number of results within the RI, are summarized in Table 3.

**Table 3:** Hematology reference interval verification results

**Analyte (unit)**	**RI partitioning**	Current RI*	**Median (IQR)**	**N**	**Results within the harmonized RI, N (%)**
RBC (×10^12^/L)	M	4.34-5.72	4.81(4.27-5.63)	20	20 (100)
	F	3.86-5.08	4.47 (4.24-4.72)	20	20 (100)
Hb (g/L)	M	138-175	144 (128-155)	20	20 (100)
	F	119-157	136 (125-139)	20	20 (100)
Hct (L/L)	M	0.415-0.530	0.416 (0.382-0.439)	20	20 (100)
	F	0.356-0.470	0.390 (0.370-0.406)	20	20 (100)
MCV (fL)	All	83.0-97.2	86.5 (84.9-88.7)	20	20 (100)
MCH (pg)	All	27.4 - 33.9	29.8 (29.1-30.6)	20	20 (100)
MCHC (g/L)	All	320-345	342 (338-344)	20	18 (90)
RDW (%)	All	9.0-15.0	12.7 (12.3-13.1)	20	20 (100)
Plt (×10^9^/L)	All	158-424	241 (217-268)	20	20 (100)
MPV (fL)	All	6.8-10.4	9.9 (9.5-10.1)	20	19 (95)
Rtc (×10^9^/L)	All	22-97	62.6 (57.2-70.7)	20	19 (95)
Rtc (Rel/10^3^ RBC)	All	5.0-21.6	12.9 (11.7-14.8)	20	19 (95)
WBC (×10^9^/L)	All	3.4-9.7	6.11 (5.43-7.15)	20	20 (100)
Neutrophils (×10^9^/L)	All	2.06-6.49	3.34 (2.72-4.24)	20	20 (100)
Neutrophils (%)	All	44-72	54.6 (49.5-61.5)	20	20 (100)
Lymphocytes (×10^9^/L)	All	1.19-3.35	2.01 (1.67-2.46)	20	20 (100)
Lymphocytes (%)	All	20-46	32.9 (26.9-38.4)	20	20 (100)
Monocytes (×10^9^/L)	All	0.12-0.84	0.55 (0.42-0.60)	20	20 (100)
Monocytes (%)	All	2-12	8.3 (7.2-9.7)	20	20 (100)
Eosinophils (×10^9^/L)	All	0.00-0.43	0.14 (0.09-0.24)	20	19 (95)
Eosinophils (%)	All	0-7	2.2 (1.3-4.0)	20	20 (100)
Basophils (×10^9^/L)	All	0.00-0.06	0.04 (0.03-0.06)	20	20 (100)
Basophils (%)	All	0-1	0.7 (0.5-0.9)	20	20 (100)
	M, < 50 y	2-13	4 (3-7)	20	20 (100)
ESR (mm/3.6 ks)	M, ≥ 50 y	3-23	9 (6-14)	20	20 (100)
	F, < 50 y	4-24	6 (5-9)	20	20 (100)
	F, ≥ 50 y	5-28	12 (9-16)	20	20 (100)
*RIs were adopted from harmonized RIs currently in use in Croatia (8). RI - reference interval. IQR - interquartile range. M - male. F - female. RBC - red blood cells. Hb - hemoglobin. Hct - hematocrit. MCV - mean cell volume. MCH - mean cell haemoglobin. MCHC - mean cell haemoglobin concentration. RDW - red cell distribution width. Plt - platelets. MPV - mean platelet volume. Rct - reticulocytes. WBC - white blood cells. ESR - erythrocyte sedimentation rate.					

### Coagulation reference intervals

All coagulation RIs are age- and sex-independent. The sample set included 10 male and 10 female individuals, aged 22 to 75 years. All tested RIs were confirmed in the first set of samples. Verification results for all analytes, including RI partitioning, current RIs, sample size, median, IQR, and the number of results within the RI, are summarized in Table 4.

**Table 4:** Coagulation reference interval verification results

**Analyte (unit)**	**RI partitioning**	Current RI*	**Median (IQR)**	**N**	**Results within the harmonized RI, N (%)**
PT (% activity)	All	≥ 0.70	1.04 (0.97 - 1.13)	20	20 (100)
APTT (s)	All	20 - 30	26 (24 - 26)	20	20 (100)
APTT (ratio)	All	0.8 - 1.2	1.0 (1.0 - 1.0)	20	20 (100)
Fbg (g/L)	All	1.8 - 3.5	2.6 (2.4 - 2.9)	20	20 (100)
*RIs were adopted from harmonized RIs currently in use in Croatia (8). RI - reference interval. IQR - interquartile range. M - male. F - female. PT - prothrombin time. APTT - activated partial thromboplastin time. Fbg - fibrinogen.					

### Clinical chemistry reference intervals

Reference intervals for total bilirubin (TBIL), direct bilirubin (DBIL), urea, aspartate aminotransferase (AST), amylase (AMY), lactate dehydrogenase (LD), lipase (Lip), potassium (K), sodium (Na), chloride (Cl), total calcium (Ca), ionized Ca, magnesium (Mg), inorganic phosphate (Phos), and CRP are age- and sex-independent. The initial sample set included 10 male and 10 female individuals, aged 22 to 75 years. All tested RIs were verified in the first set of samples, except for total Ca. A second set of 20 samples, including 10 male and 10 female individuals aged 23 to 73 years, was analyzed for total Ca, but the verification criteria were still not met.

Reference intervals for albumin (Alb), total protein (TP), and glucose (Glc) are age-dependent. Due to an insufficient number of participants, verification of RIs for Alb and TP in individuals over 70 years of age was not performed. The sample set for verification of RIs between 20 and 70 years included 10 male and 10 female individuals, aged 22 to 63 years. Both tested RIs were verified in the first set of samples. The sample sets for Glc RIs included 10 male and 10 female individuals in each age group: 22 to 29 years and 30 to 75 years. Both RIs were verified in the first set of samples.

Reference intervals for creatinine (CREA), uric acid, alanine aminotransferase (ALT), gamma-glutamyltransferase (GGT), CK, iron (Fe), unsaturated iron-binding capacity (UIBC), and total iron-binding capacity (TIBC) are sex-dependent. The male sample set included individuals aged 26 to 75 years, and the female sample set included individuals aged 22 to 68 years. All tested RIs were verified in the first set of samples.

Reference intervals for alkaline phosphatase (ALP) are both age- and sex-dependent. The first sample set included male individuals aged 26 to 75 years, female individuals aged 22 to 46 years, and female individuals aged 52 to 68 years. In this set, the RIs for males of all ages and for females under 50 years of age were not verified, while the RI for females over 50 years was confirmed. A second set of 40 samples was collected, consisting of 20 male individuals aged 28 to 73 years and 20 female individuals aged 22 to 49 years; however, the verification criteria still were not met.

Verification results for all analytes, including RI partitioning, current RIs, sample size, median, IQR, and the number of results within the RI, are summarized in Table 5.

**Table 5:** Clinical chemistry reference interval verification results

**Analyte (unit)**	**RI partitioning**	Current RI*	**Median (IQR)**	**N**	**Results within the harmonized RI, N (%)**
TBIL (µmol/L)	All	3-20	13 (10-15)	20	20 (100)
DBIL (µmol/L)	All	< 5	2 (1-3)	20	20 (100)
Glc (mmol/L)	All; < 30 y	4.2-6.0	4.5 (4.3-4.8)	20	20 (100)
	All; ≥ 30 y	4.4-6.4	4.7 (4.5-4.9)	20	19 (95)
CREA^†^ (µmol/L)	M	64-104^†^	85 (82-94)	20	20 (100)
	F	49-90^†^	69 (64-77)	20	20 (100)
Uric acid (µmol/L)	M	182-403	326 (298-346)	20	19 (95)
	F	134-337	252 (237-275)	20	20 (100)
Urea (mmol/L)	All	2.8-8.3	5.4 (4.1-6.2)	20	20 (100)
AST (U/L)^‡^	All	11-34^‡^	23 (20-26)	20	20 (100)
ALT (U/L)^‡^	M	9-59^‡^	26 (22-31)	20	20 (100)
	F	8-41^‡^	19 (16-24)	20	20 (100)
AMY (U/L)	All	23-91	63 (52-70)	20	20 (100)
	M	60-142	70 (60-78)^§^	40	15 (75)^§^
			68 (53-73)^║^		13 (65)^║^
ALP (U/L)	F; < 50 y	54-119	54 (40-67)^§^	40	10 (50)^§^
			55 (40-66)^║^		11 (55)^║^
	F; ≥ 50 y	64-153	77 (72-96)	20	19 (95)
GGT (U/L)	M	11-55	28 (18-34)	20	20 (100)
	F	9-35	17 (14-20)	20	20 (100)
CK (U/L)	M	< 177	109 (69-130)	20	20 (100)
	F	< 153	81 (67-113)	20	20 (100)
LD (U/L)	All	< 241	166 (156-179)	20	20 (100)
Lip (U/L)	All	13-60	19 (16-23)	20	19 (95)
K (mmol/L)	All	3.9-5.1	4.2 (4.1-4.4)	20	20 (100)
Na (mmol/L)	All	137-146	141 (140-141)	20	20 (100)
Cl (mmol/L)	All	97-108	104 (103-105)	20	20 (100)
Ca, total (mmol/L)	All	2.14-2.53	2.46 (2.38-2.53)^§^	40	15 (75)^§^
			2.47 (2.40-2.52)^║^		16 (80)^║^
Ca, ionized (mmol/L)	All	1.18-1.32	1.25 (1.23-1.27)	20	19 (95)
Mg (mmol/L)	All	0.65-1.05	0.83 (0.81-0.85)	20	20 (100)
Phos (mmol/L)	All	0.79-1.42	1.17 (1.08-1.23)	20	19 (95)
Fe (µmol/L)	M	11-32	21 (16-25)	20	20 (100)
	F	8-30	15 (13-19)	20	20 (100)
UIBC (µmol/L)	M	25-54	39 (34-44)	20	19 (95)
	F	26-59	46 (39-53)	20	20 (100)
TIBC (µmol/L)	M	49-72	60 (57-64)	20	20 (100)
	F	49-75	64 (55-66)	20	20 (100)
Alb (g/L)	All, < 70 y	40.6-51.4	45.5 (43.8-49.1)	20	19 (95)
	All, ≥ 70 y	39.6-48.4	NA	2	NA¶
CRP (mg/L)	All	< 5.0	0.9 (0.5-1.7)	20	20 (100)
TP (g/L)	All, < 70 y	66-81	72 (70-76)	20	19 (95)
	All, ≥ 70 y	66-80	NA	2	NA¶
*RIs were adopted from harmonized RIs currently in use in Croatia (8), unless otherwise specified. ^†^RIs according to reference (12). ^‡^RIs according to reference (13). ^§^First set of samples. ^║^Second set of samples. **^¶^**RIs for individuals aged ≥ 70 years were not verified due to an insufficient number of reference individuals in this age group. RI - reference interval. IQR - interquartile range. M - male. F - female. TBIL - bilirubin, total. DBIL - bilirubin, direct. Glc - glucose. CREA - Creatinine. AST - aspartate aminotransferase. ALT - alanine aminotransferase. AMY - amylase. ALP - alkaline phosphatase. GGT - gamma-glutamyltransferase. CK - creatine kinase. LD - lactate dehydrogenase. Lip - lipase. K - potassium. Na - sodium. Cl - chloride. Ca - calcium. Mg - magnesium. Phos - inorganic phosphate. Fe - iron. UIBC - unsaturated iron-binding capacity. TIBC - total iron-binding capacity. Alb - albumin. CRP - C-reactive protein. TP - total protein.					

**Figure 1:**
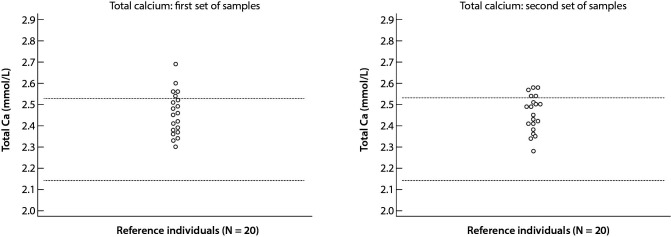
Distribution of total calcium (Ca) results relative to the evaluated reference interval in the first and second set of samples. Individual results are shown as dots. Dashed horizontal lines represent the lower and upper reference limits.

**Figure 2:**
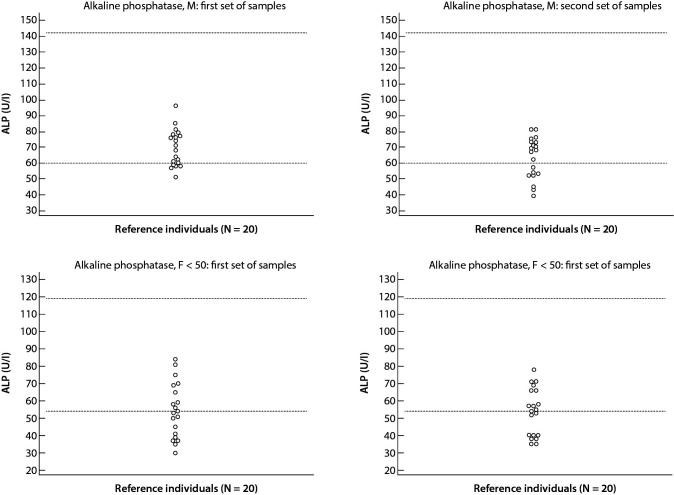
Distribution of alkaline phosphatase (ALP) results relative to the evaluated reference interval in the first and second set of samples for males of all ages (M) and females under 50 years of age (F < 50). Individual results are shown as dots. Dashed horizontal lines represent the lower and upper reference limits.

The verification results for total Ca and ALP in males of all ages and in females under 50 years of age did not meet verification criteria in either sample set. For total Ca, all out-of-range results were observed above the upper reference limit, whereas for ALP, all out-of-range results were below the lower reference limit. The distribution of total Ca and ALP results relative to the applied RIs is illustrated in Figures 1 and 2. Further verification was performed using RIs from alternative literature sources and RIs provided by the reagent manufacturer (Beckman Coulter). For total Ca, RIs from the UK Pathology Harmony harmonization project were verified, while for ALP, RIs from the IFCC primary reference procedure were verified (*18*, *19*). Verification using both data sets met the predefined verification criteria for both analytes for RIs derived from harmonized literature sources, as well as those provided by the reagent manufacturer. Results for all data sets are presented in Table 6.

**Table 6:** Alternative sources reference interval verification results for total calcium and alkaline phosphatase

			**International harmonized RI**		**Reagent** **manufacturer RI**	
**Analyte (unit)**	**RI partitioning**	**Median (IQR)**	**RI**	**Results within the RI, N (%)**	**RI**	**Results within the RI, N (%)**
Ca, total (mmol/L)	All	2.46 (2.38-2.53)^‡^	2.20-2.60*	19 (95)	2.20-2.65	19 (95)
		2.47 (2.40-2.52)^§^		20 (100)		20 (100)
	M	70 (60 - 78)^‡^	43-115^†^	20 (100)	43-115	20 (100)
		68 (53 - 73)^§^		19 (95)		19 (95)
ALP (U/L)	F; < 50 y	54 (40 - 67)^‡^	33-98^†^	19 (95)	33-98	19 (95)
		55 (40 - 66)^§^		20 (100)		20 (100)
	F; ≥ 50 y	77 (72 - 96)	NA^║^	NA^║^	NA^║^	NA^║^
*RIs according to reference (18). ^†^RIs according to reference (19). ^‡^First set of samples. ^§^Second set of samples. ^║^Verification of RIs for females aged ≥ 50 years was not performed because no literature-based (19) or manufacturer-provided RIs are available for this age group. RI - reference interval. IQR - interquartile range. M - male. F - female. Ca - calcium. ALP - alkaline phosphatase.						

## Discussion

The results of this study indicate that the harmonized RIs recommended by the CCMB for hematology, coagulation, and clinical chemistry laboratory tests are suitable for the population of apparently healthy volunteers from one clinical hospital center. The findings suggest that the existing harmonized RIs, derived from a population with demographic characteristics comparable to those of our study cohort, remain clinically relevant despite methodological and population changes in recent years. However, the recommended Croatian harmonized RIs for total Ca and ALP could not be verified in this study. Harmonized RIs from alternative literature sources were considered for further verification, including RIs for total Ca from the UK Pathology Harmony project and IFCC-recommended RIs for the IFCC traceable method for ALP determination. The RIs from these two literature sources were verified, confirming the high degree of standardization and harmonization of both methods. Differences in verification outcomes between previously recommended and alternative harmonized RIs may reflect differences in RI derivation methodology and reference population characteristics. This highlights the relevance of RI origin and derivation methodology when interpreting verification results. In addition to international harmonized RIs, manufacturer-provided RIs for total Ca and ALP were also evaluated during the verification process, in accordance with routine laboratory practice. Both the first and the second verification sets fulfilled the manufacturer-recommended RIs for these analytes. Although manufacturer RIs may be applied when appropriate and are commonly used in the absence of published reference data, they represent the lowest level of currently available evidence, as they are typically derived from limited or insufficiently described reference populations. In contrast, harmonized RIs proposed by international initiatives or professional societies are based on systematically designed studies, transparent methodologies, and broader population data, representing a higher level of scientific evidence (*1*, *6*). It should be noted that the manufacturer-recommended RIs for ALP were identical to those proposed by international harmonized sources (*19*). However, the original harmonized publication was preferred, as it provided detailed information on study design and population characteristics, which are not available in reagent package inserts. On this basis, harmonized RIs from international literature were considered more appropriate for routine use and verification. The fact that not all RIs were verified highlights the need for periodic review, verification, and revision of RIs, which is also one of the requirements of the CLSI EP28-A3c guidelines and the ISO standard 15189:2022 (*12*, *16*). However, neither CLSI nor ISO standards define a fixed time interval for RI verification. Instead, RI verification should be performed when relevant changes occur that may affect the applicability of existing RIs (*16*). Such changes include modifications of analytical methods or platforms, reagent reformulations, changes in calibration or traceability, or significant changes in the demographic or clinical characteristics of the reference population. In addition, RI verification is recommended when introducing new tests or when clinically relevant discrepancies between patient results and expected distributions are observed (*1*, *12*, *16*).

This study has several limitations, including the selection and size of the study population and the analytical methods used. The population size, which included 20 individuals per subgroup for each RI, is sufficient for the verification of RIs according to the CLSI EP28-A3c guidelines. However, this number is insufficient for *de novo* establishment of RIs should such a need arise. For this reason, in cases where the harmonized RIs currently recommended in Croatia were not successfully verified, international harmonized RIs were adopted instead of establishing *de novo* RIs. An additional limitation relates to the statistical nature of RI verification. The pass/fail outcome of RI verification depends on the number of tested reference individuals and assumes independence between individual analytes. However, some laboratory parameters are biologically and analytically correlated, such as RBC indices, liver enzymes, and TP and Alb. Therefore, some non-verified RIs should be interpreted with caution, particularly when biologically related analytes are considered. Additionally, the age distribution of the study population did not permit verification of age-partitioned RIs for individuals over 70 years (for Alb and TP), limiting conclusions for that demographic. The study population consisted of apparently healthy volunteers, including hospital staff and individuals from the general population. However, the population was relatively homogeneous, as all participants were affiliated with a single clinical hospital center. Due to this homogeneity, there is a possibility that the selected population does not fully represent the biological variability of the broader Croatian population. This may limit the generalizability of the obtained results to other regions of the country with potentially different demographic characteristics. Regarding analytical methods, hematology parameters, ESR, coagulation assays, clinical chemistry tests, and ionized Ca were each measured on a single analytical platform per test group (Sysmex XN1000 for hematology, iSED for ESR, Sysmex CS2500 for coagulation, Beckman Coulter DxC700AU for clinical chemistry, and Radiometer ABL90 FLEX for ionized Ca). This may limit the applicability of the results to laboratories using other analytical systems. These limitations highlight the importance of conducting multicenter studies with larger and more diverse populations, as well as cross-platform analytical comparisons, in order to ensure the broader applicability of verified RIs across different laboratory settings and regions.

In conclusion, our findings support the continued use of most harmonized RIs currently recommended in Croatia for adults over the age of 20, while also emphasizing the importance of regular RI verification, especially in light of changing demographic and methodological conditions.

## Data Availability

The data generated and analyzed in the presented study are available from the corresponding author on request.
